# Training of Motion Control May Not Improve Tool-Manipulation Ability in Rats (*Rattus norvegicus*)

**DOI:** 10.3389/fpsyg.2022.931957

**Published:** 2022-07-13

**Authors:** Akane Nagano

**Affiliations:** ^1^Laboratory for Imagination and Executive Functions, RIKEN Center for Brain Science, Wako, Japan; ^2^Japan Society for the Promotion of Science, Chiyoda-ku, Japan

**Keywords:** rats, rodents, tool-use behavior, tool-manipulation, motion control

## Abstract

In recent times, previous studies have reported the manipulation of tools by rats and degus in controlled experimental settings. However, a previous study reported that only one out of eight experimentally naïve rats could manipulate a rake-shaped tool according to the position of a food reward without prior experience of obtaining the reward with the tool before the test. The present study aimed to improve the training of rats and investigate rodents’ ability to manipulate tools according to food position. Stricter criteria were employed when training the rats to promote the rats’ monitoring of their own tool manipulation. Additional training was introduced to give them the opportunity to learn that the reward moved closer to them by pulling an object connected to the reward. The present study showed that only one of eight rats could manipulate a tool according to the position of the reward without prior experience of obtaining the reward with the tool or perceiving that part of the tool came in contact with the reward, as the previous study showed. The change in training did not enhance the rats’ tool-manipulation ability according to the food position. These procedures should be conducted in a wider variety of animals to investigate whether the training in motion control can promote the subjects’ effective tool-use behavior.

## Introduction

Tool-use behavior in non-human animals has been investigated mainly in primates and birds (Bentley-Condit and Smith, 2010). The reason is that these animals have relatively high visual acuity, so it is easier to test their tool-use behavior than it would be in low-visual-acuity animals (Shumway, 2008). St Amant and Horton (2008, p. 1203) proposed the definition of tool-use: “Tool-use is the exertion of control over a freely manipulable external object (the tool) with the goal of (1) altering the physical properties of another object, substance, surface, or medium (the target, which may be the tool user or another organism) via a dynamic mechanical interaction, or (2) mediating the flow of information between the tool user and the environment or other organisms in the environment.” In non-human primates, previous studies have reported that monkeys could be trained to manipulate a rake-shaped tool to obtain food beyond their reach in situations in which they could not obtain the food by pulling the tool perpendicularly to themselves via a step-by-step protocol [e.g., Japanese macaques (*Macaca fuscata*): Yamazaki et al., 2009; common marmosets (*Callithrix jacchus*): Yamazaki et al., 2011]. The distance between the rake and reward was gradually extended in the training used in these previous studies. In birds, a Goffin’s cockatoo (*Cacatua goffiniana*) spontaneously manufactured a stick tool from a European larch and used it to rake food beyond its reach, but the other two cockatoos did not use tools to obtain the food (Auersperg et al., 2012).

Many researchers have attempted to shed light on the evolutionary processes of physical causal understanding by conducting tool-use tasks with a variety of animal species (Bentley-Condit and Smith, 2010). Primates, including humans, emerged through divergence of evolutionary processes from a common ancestor of mammals to rodents (Krubitzer, 2009). Thus, investigations of tool-use behavior in rodents, which have shared evolutionary processes with humans, are essential to shed light on the evolutionary processes of physical causal understanding in humans.

Recent studies have investigated tool-use behavior in relatively low-visual-acuity rodents (Prusky et al., 2002), including rats (*Rattus norvegicus*) (Nagano and Aoyama, 2017a,b; Nagano, 2019a,b, 2021) and degus (*Octodon degus*) (Okanoya et al., 2008; Kumazawa-Manita et al., 2013) in controlled experimental settings. Previous studies on rats have reported that these rodents manipulated a rake-shaped tool based on the position of the food reward placed beyond their reach after undergoing tool-use training (Nagano and Aoyama, 2017b; Nagano, 2019b). In this test, the rats could use only the position of the reward in relation to the rake as a cue to manipulate the rake effectively, and could not obtain the reward just by pulling the rake perpendicularly to themselves because the rake was placed at the center of the experimental apparatus, and the reward was randomly placed on either the left or right side of the rake. However, the rats could manipulate the rake in the direction of the reward by using a strategy similar to that learned during training because they had the experience of obtaining the reward by using tools (Nagano and Aoyama, 2017b; Nagano, 2019b). In contrast, Nagano (2021), who used the same test implemented in the two previous studies (Nagano and Aoyama, 2017b; Nagano, 2019b), reported that a rat manipulated the rake according to the reward position without prior experience of obtaining the reward with the tool or perceiving that a part of the tool came in contact with the reward that might cause it to move. However, only one out of the eight rats could manipulate the rake according to the position of the reward (Nagano, 2021). Thus, it could not be concluded that rats have a primitive ability to manipulate tools according to food position. In the main training (rake-manipulation training) by Nagano (2021), the rats were trained to move the rake laterally over successively greater distances without any food reward placed on the experimental apparatus. This training differed from the previous studies (Nagano and Aoyama, 2017b; Nagano, 2019b), particularly in that it promoted the manipulation of the rake in the lateral direction. Additionally, the rats were trained to obtain a reward on the apparatus directly with their paws or mouth (food-obtaining training).

The test mentioned above, where the rake was placed at the center and the food reward placed on either the left or right side of the rake, followed the food-obtaining training. Nagano (2021) suggested improving the methods for rake-manipulation and food-obtaining training as follows: the rake-manipulation training should employ stricter criteria to promote the rats’ monitoring of their own manipulation of the rake, and the rats should be offered the experience of pulling something (i.e., thread sewn into the food reward in the present study) to obtain the reward. This incorporates the suggestions of Connolly and Dalgleish (1989) and Ramsey et al. (2021) that visual monitoring is necessary for tool-manipulation. Moreover, Nagano (2021) did not confirm whether their low visual acuity was the cause of their low performance in the test. In the study, the rats could obtain the reward just by moving the rake laterally over a certain distance in the training (Nagano, 2021). Therefore, it is possible that the rats could not manipulate the rake in the direction of the reward due to the awkwardness of their paws movements in the test of this previous study, not due to their low visual acuity (Nagano, 2021). Therefore, further confirmation is required to discount the possibility that most of the rats could not manipulate the rake based on the position of the reward due to their low visual acuity.

The present study aimed to improve the training techniques applied in Nagano (2021) as well as to investigate the effect of this enhanced training technique on the ability of rodents with low-visual acuity to manipulate tools according to food position without prior experience of obtaining rewards using the tool. In the rake-manipulation training in the present study, stricter criteria for success were employed to promote the rats’ monitoring than those in Nagano (2021). Focusing on both the motion of their own paws and the criteria that exist in the external environment may promote them to pay attention to the relationship between their own motion and the external objects (the tool and food rewards) in the tool-use situations. In addition, another kind of training was introduced instead of the food-obtaining training, in which the rats were trained to pull a thread fastened to a food reward or a thread fastened to no reward. One of the purposes of this training was to promote the rats to pay attention to the position of the reward in the positional discrimination test. The rats never perceived that one object collided with another object and sent it into motion in the food-obtaining training. I hypothesized as follows: if trainings of motion control improve the tool-manipulation ability in rats, better performances by the rats would be observed in this test than in those of Nagano (2021).

A raking tool was used for the behavioral task for rats in the present study because the previous studies have reported that degus and rats could use rake-shaped tools (Kumazawa-Manita et al., 2013; Nagano and Aoyama, 2017a,b; Nagano, 2019a,b, 2021), and the animals in the present study were expected to learn to use the raking tool in a shorter period because it is easier for them to handle the tool with their forelimbs. Tool-use behavior in rats in the wild has never been reported (Bentley-Condit and Smith, 2010), and spontaneous tool-use behavior has never been observed in the experimental settings (Nagano and Aoyama, 2017a,b; Nagano, 2019a,b, 2021). However, rats are skillful in using their forelimbs dexterously to pull strings to obtain a food (Blackwell et al., 2018). The movement consists of alternating forelimb movements in which a limb is advanced to grasp a string and withdraw it toward the body in order to retrieve a food reward. The movements of aim, advance, grasp, pull and push are associated with hand shape changes including collect, overgrasp, grasp and release (Blackwell et al., 2018). Irvine et al. (2010) have also reported that rats handle objects with their forelimbs spontaneously and dexterously.

## Methods

### Animals

Eight experimentally naïve three-month-old male Brown-Norway rats (subject numbers: BN57–BN64; Shimizu, Kyoto, Japan) were individually housed in wire cages. On the last day of free feeding, the rats weighed an average of 273.38 g (*SD* = 6.93). During training and testing, rats were maintained at around 85% of their free feeding weight. However, all rats could gain approximately 10 g/month. The animal room was maintained under a 12-h light/dark cycle (light phase: 8:00–20:00). All training and testing sessions were conducted during the light phase. All procedures and treatments were approved by the Doshisha University Animal Experiment Committee (protocol number: A17051), and were conducted in accordance with guidelines established by the Doshisha University Ethics Review Committee.

### Apparatus

Experiments were performed in an experimental box identical to the one used in the previous study on rats (Nagano, 2021) (see Supplementary Material for details). The sliding doors were mounted on the front of the box. One of two kinds of sliding doors (one without a hole and one with a square hole) was always placed in front of the box. The door had a square hole in its upper portion and was used to offer rewards to the rats by hand. An experimental board, on which the tool and reward were presented, was set in front of the door. Black lines were drawn in a square lattice on the board.

**FIGURE 1:**
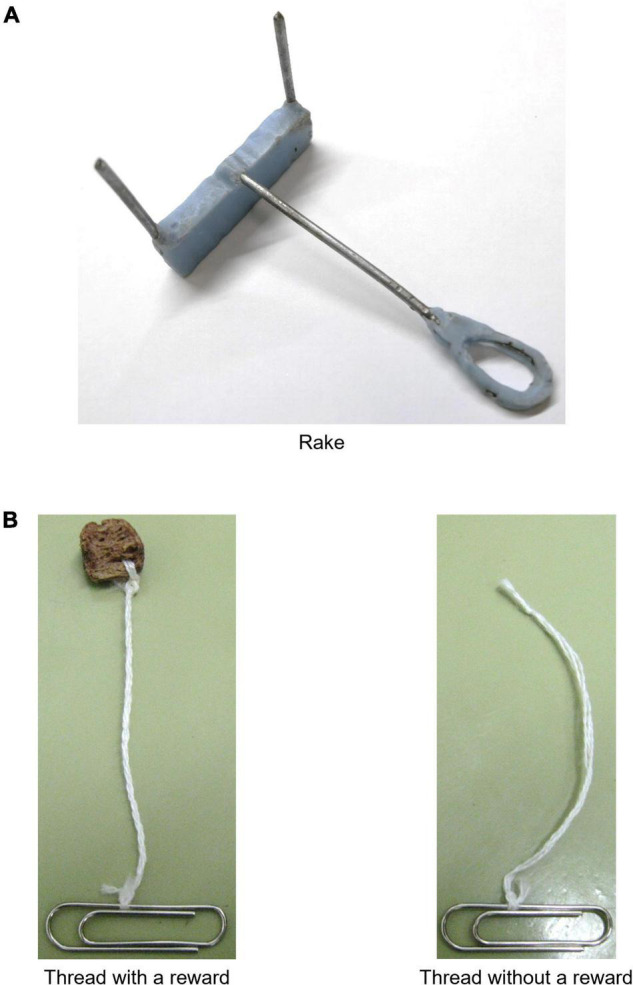
Rake and threads. **(A)** Rake used in the rake-pulling training, rake-manipulation training, and positional discrimination test. **(B)** Examples of threads with a reward and without a reward used in the thread-pulling training. One end was sewn to the reward for threads with a reward or tied into a knot for threads without a reward. The other end of both types of threads was tied to a gem clip.

The tool was identical to the one used in the previous study (Nagano, 2021) (Figure 1A, see Supplementary Material for details). The rake-shaped tool had a rectangular blade and a wire handle. In addition, two kinds of threads (one with a reward and one without a reward) were used in the training immediately before the test (Figure 1B, see Supplementary Material for details). Three-hundred and four threads were used for each kind of thread (608 threads in total). For threads with a reward, one end of each strand was sewn to a piece of cereal, and the other end was tied to a gem clip. For threads without a reward, one end of each strand was tied into a knot without a reward, and the other end was tied to a clip.

### Procedure

#### Training

**FIGURE 2:**
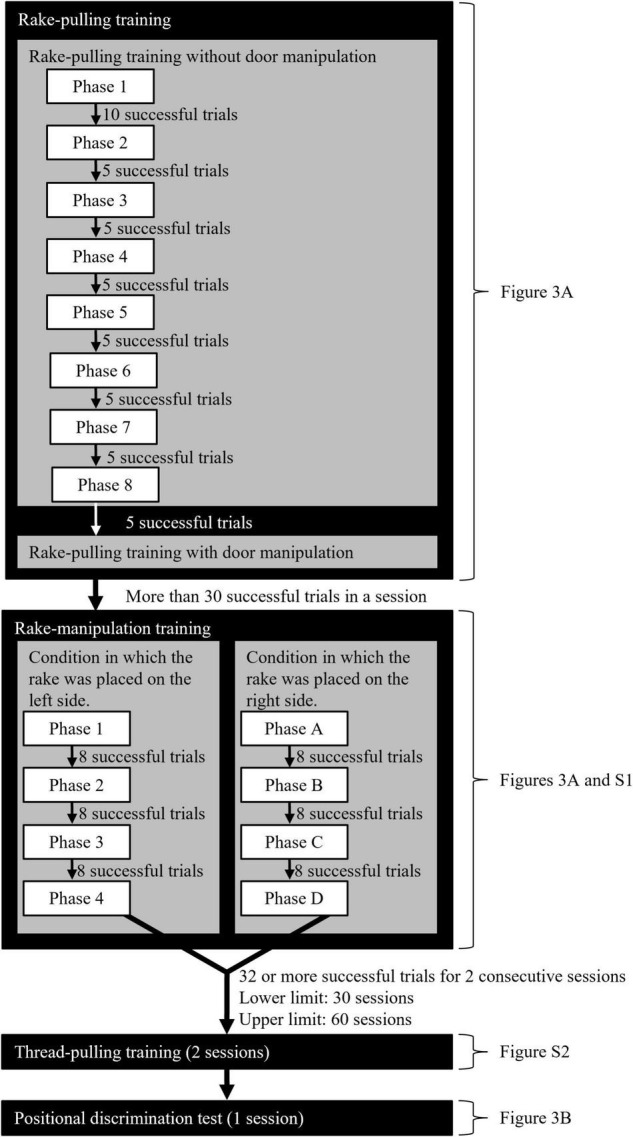
Flowchart of the rake-pulling, rake-manipulation, and thread-pulling trainings and the positional discrimination test. Each daily experimental session consisted of 40 trials, except the thread-pulling training, which had 38 trials.

The training consisted of rake-pulling training, rake-manipulation training, and thread-pulling training (Figure 2, see Supplementary Material for details). Each daily experimental session consisted of 40 trials, with the exception of the thread-pulling training, for which a session consisted of 38 trials. A piece (one-eighth to one-sixth) of chocolate-flavored loop cereal was used as a food reward in each trial.

#### Rake-Pulling Training

The rake-pulling training procedure in the present study was similar to that in Nagano (2021). The experimenter presented the rake-shaped tool on the experimental board without any food reward, and the rats first learned to pull the rake to the end of the experimental board (i.e., toward the experimental box). The rake was alternately placed on either side.

**FIGURE 3:**
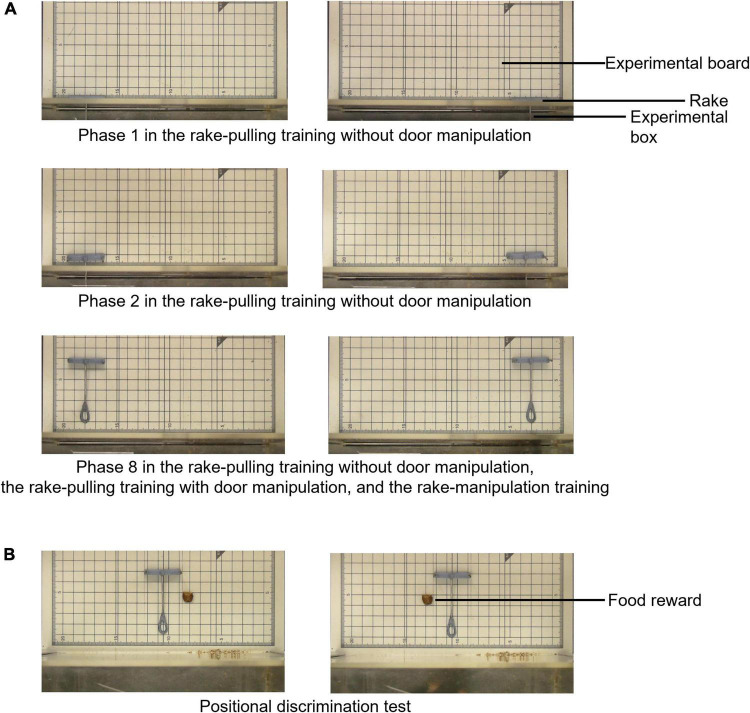
Arrangement of the rake and reward during the rake-pulling training with or without door manipulation, thread-pulling training, and positional discrimination test. **(A)** Arrangement in Phases 1, 2, and 8 of the rake-pulling training without door manipulation, rake-pulling training with door manipulation, and rake-manipulation training. Only the rake was placed on the left or right side of the experimental board, and the reward was not placed on the board during the training. The rake’s position in Phase 8 of the rake-pulling training without door manipulation and rake-pulling training with door manipulation was the same as that during the rake-manipulation training. **(B)** Arrangement during the positional discrimination test. The rake was placed at the center of the experimental board, and the reward was placed on the left or the right side of the rake.

This training was divided into eight phases (Figures 2, 3, and see Supplementary Material for details), and the criterion for reward obtainment gradually became stricter. At the beginning of Phase 1, the experimenter placed the rake so that the distance from the door of the experimental box was 0 cm, and offered a reward when the rat touched the rake with either its paw, nose, or mouth (successful trial). The experimenter offered a reward by hand through the small hole in the sliding door in successful trials (Supplementary Video 1). In Phases 2 to 8, the distance between the blade of the rake and the door was increased by 1.0 cm in each phase (Figure 3A).

#### Rake-Manipulation Training

In the rake-manipulation training, the rats were trained to move the rake laterally within fixed ranges (Figure 2 and Supplementary Figure 1). In Nagano (2021), a trial was considered a success when the rat pulled the rake so that either of the wires glued to the rake blade touched the door while the wire of the rake blade passed over a criterion line in each phase. In the present study, the criterion for a successful trial was made stricter by narrowing the range that the rake blade had to be brought to. Apart from this alternation, the used procedure followed that in Nagano (2021).

In this training, the same procedure was used as in the rake-pulling training, except that the criterion for a successful trial became stricter. The criterion ranges were set to 5.0-mm intervals to the right of the rake when the rake was placed on the left side of the experimental board from the rat’s perspective (Supplementary Figure 1A). The criterion ranges in each phase became narrower toward the center of the board every time the rat attained each criterion (Supplementary Figure 1). The four criterion ranges corresponded to the four phases: 25.0 mm for Phase 1 and 10.0 mm for Phase 4. This arrangement was mirrored when the rake was placed on the right side of the experimental board, where criterion ranges for Phases A to D corresponded to those for Phases 1 to 4, respectively (Supplementary Figure 1B). In each phase, once the rat pulled the rake so that either of the wires glued to the rake blade touched the door while the wire of the rake blade that was close to the center of the board was positioned within the criterion range, the experimenter retrieved the rake and offered the rat a reward through the hole in the door (successful trial, Supplementary Video 2); when the wire of the rake blade closer to the center of the board did not fall within the criterion range, the experimenter retrieved the rake without offering a reward (failed trial, Supplementary Video 3). Thus, the rake and reward were never presented simultaneously. Through this procedure, the rats were trained to laterally manipulate within gradually narrower ranges. The rake-manipulation training continued until the rat achieved the criterion of the last phase under both arrangement conditions (Phases 4 and D) and succeeded in 32 or more trials for two consecutive sessions.

#### Thread-Pulling Training

In the thread-pulling training, the rats were trained to pull the thread with or without a reward with their paws or mouth (Figure 2, Supplementary Video 4). The first purpose of the training was to offer the rats opportunities to learn that the reward perceived through the sliding door was identical to the reward obtained by the rat in the experimental box, and that the reward was moved toward the rats by pulling an object (the gem clip tied by the embroidery thread). The second purpose of the training was to confirm that they could identify the position of the reward placed at a distance similar to that in the subsequent test. The thread was never removed from the reward on the experimental board, and therefore the rats were not meant to realize that any hard object (part of the tool) was in contact with the reward at all. The sliding door without holes was used. The daily experimental sessions consisted of 38 trials.

#### Positional Discrimination Test

After the thread-pulling training, the positional discrimination test was performed for one session according to a procedure similar to that used in previous studies on rats (Nagano and Aoyama, 2017b; Nagano, 2019b, 2021) (Figure 2). In this test, the experimenter examined whether the rats could manipulate the rake laterally in relation to the position of food, even when the tool and food were presented simultaneously for the first time. The rake was placed at the center of the experimental board, and the reward was placed on either the left or right side of the rake (Figure 3B). The rats could obtain the reward when they pulled the rake in relation to the position of the reward until they pulled the rake to the position of the reward in the vertical direction (Supplementary Video 5). The rats were required to move the rake laterally before pulling it to obtain the reward.

### Data Analyses and Statistical Methods

Rat behavior was analyzed using video records from the training and the positional discrimination test. The statistical analyses were performed using SPSS Statistics version 25.0. The criterion for statistical significance was set at *p* < 0.05.

The manipulation direction of the rake was analyzed using the method used in previous studies on rats (Nagano and Aoyama, 2017b; Nagano, 2019b, 2021). In this test, when the rat manipulated the rake toward the reward, it was recorded as a correct-direction trial. In contrast, when the rat manipulated the rake away from the reward, it was recorded as an incorrect-direction trial. These determinations were based on whether the intersection point of the blade and the handle was on the left or right side of the center line of the experimental board when the rat pulled the rake 2.0 cm (i.e., to the horizontal line contacting the reward). The correct-direction rate is a behavioral index that would enable the detection of trials in which the subject understood the appropriate direction to move the tool to obtain the reward, but does not successfully manipulate the rake because of insufficient motor ability (Nagano and Aoyama, 2017b). Trials in which the rat did not pull the rake, stopped pulling it before pulling it 2.0 cm toward itself, or flipped the rake out of reach before pulling it 2.0 cm toward itself were excluded from this analysis (BN57: seven trials; BN58: 18 trials; BN59: 19 trials; BN60: one trial; BN61: one trial; BN62: zero trial; BN63: one trial; BN64: zero trial).

In addition, the position of each rat’s nose was analyzed when it first touched the rake with the left or right paw in each trial based on the video recordings from the positional discrimination test. This analysis was conducted to investigate whether the position of the rat in relation to the rake and the reward influenced their correct-direction rate by using the method used in previous studies on rats (Nagano and Aoyama, 2017b; Nagano, 2019b, 2021). For this analysis, the first column on the experimental board was divided into 21 areas (Areas 1–21) based on the squares on the board (Supplementary Figures 3, 4), and the position of the rat’s nose was recorded after the trial began. The position of each rat’s nose was determined from the video frame at the moment the rat first touched the rake.

To analyze the relationships between the position of rats’ noses and the correct-direction rates in the positional discrimination test, the first column (Areas 1–21) of the experimental board was divided into the area to the left (Areas 1–10) and right (Areas 12–21) of the rake handle, with Area 11 at the center (Supplementary Figures 3, 4). The number of trials in which the rat’s nose was in the left or right side area at the time of the first touch on the rake was calculated for each trial. Moreover, when the reward was placed left of the rat’s view (20 out of 40 trials), if the rat’s nose was in the left side area, then it was recorded as an ipsilateral trial. Under the same conditions, if the rat’s nose was in the right side area, then it was recorded as a contralateral trial. Similarly, when the reward was placed right of the rat’s view (20 out of 40 trials), if its nose was in the right side area, then it was recorded as an ipsilateral trial; but if the rat’s nose was in the left side area, then it was recorded as a contralateral trial. Trials in which rats’ noses were located in the center (Area 11) of the board when they first touched the rake were not considered ipsilateral or contralateral.

For the rake-manipulation training, the daily success rates were calculated by dividing the number of trials in which each rat moved the rake laterally within the criterion range within 60 s (number of successful trials) by the total number of trials (40 trials per day).

In the thread-pulling training, the average thread-pulling rates per session were calculated for the threads with and without a reward separately. The thread-pulling rates were calculated by dividing the number of trials in which each rat pulled the thread (with or without a reward) to the position in which the reward or knot entered in the box (number of thread-pulling trials) by the total number of trials in each thread condition (19 trials per session). A two-way analysis of variance (ANOVA) was performed with thread type and session as within-subject factors, followed by simple main effect analyses. In addition, the average thread-contacting rates per session were calculated for the threads with a reward and threads without a reward separately. The thread-contacting rates were calculated by dividing the number of trials in which the rat touched the rake with its paws or mouth within 60 s (number of thread-contacting trials) by the total number of trials in thread condition (19 trials per session). A two-way ANOVA was performed with thread type and session as within-subject factors followed by simple main effect analyses.

The success rate was also calculated for each rat in the positional discrimination test by determining whether the rats manipulated the rake toward the reward (in the correct-direction) or not (in the incorrect-direction), and comparing the number of trials in which the rake was moved in either direction (40 trials). The correct-direction rate was calculated by dividing the number of trials in which each rat manipulated the rake toward the reward (number of correct-direction trials) by those in which the rat manipulated the rake in either direction. The correct-direction trials included trials in which the rat manipulated the rake in the correct-direction but failed to obtain the reward. Using binomial tests, the number of correct- and incorrect-direction trials was compared for each rat. In the binomial tests, the null hypothesis was that the correct-direction rate was 50%. In addition, data analysis showed whether each rat manipulated the rake in the correct-direction from the beginning of the session in the test. The daily sessions (40 trials) were divided into eight blocks to calculate the average correct-direction rate of rake manipulation, with each block consisting of five trials. Trials 31–35 in one rat (BN58) and Trials 26–40 in another (BN59) were excluded from this analysis because the individuals never pulled the rake in these trials.

Ipsilateral trials were calculated for each rat to analyze the relationship between the position of the rats’ noses and the correct-direction rates in the positional discrimination test. The number of ipsilateral and contralateral trials was compared for each rat using two-tailed binomial tests.

## Results

Results from the rake-pulling, rake-manipulation, and thread-pulling training are described in Supplementary Material.

### Positional Discrimination Test

**FIGURE 4:**
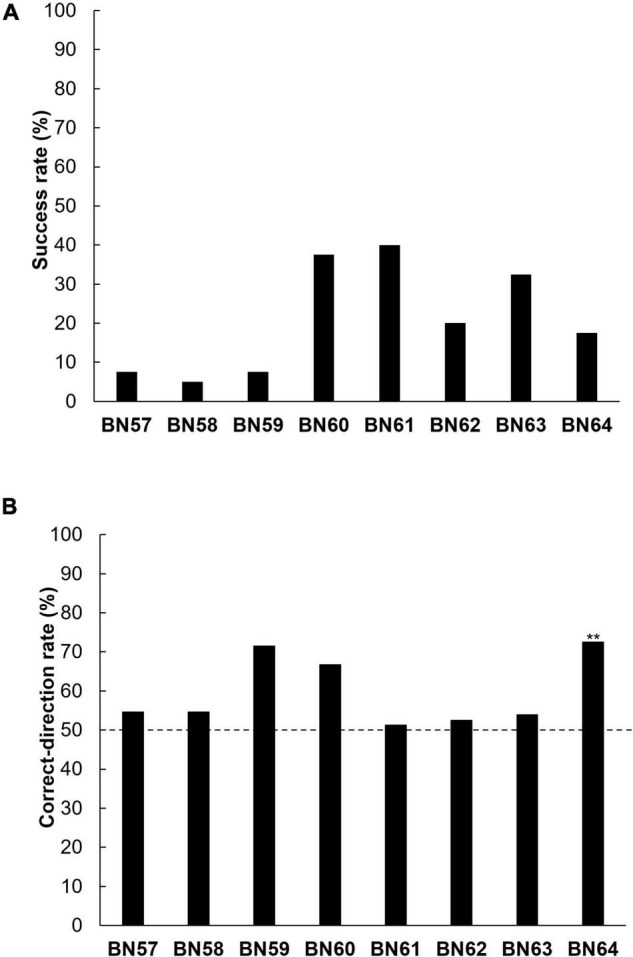
Individual (BN57–BN64) performance in the positional discrimination test. **(A)** Individual success rates. **(B)** Individual correct-direction rates. The broken line indicates the level of chance (^**^ *p* < 0.01).

The success rates in the positional discrimination test were low in all the rats (5.0–40.0%, Figure 4A). One of the eight rats (BN64) manipulated the rake in the correct-direction significantly more frequently than in the incorrect-direction (BN57–BN63: *n. s.*, BN64: *p* < 0.01, binomial tests, Figure 4B). In addition, the performance of the one rat (BN64) that manipulated the rake in the correct-direction the most did not show a trend toward improvement in its correct-direction rate within a session (Supplementary Figure 8).

The position of each rat’s nose the first time it touched the rake with the left or right paw in each trial was analyzed in the positional discrimination test (Supplementary Figures 3, 4). The purpose of this analysis was to investigate the possibility that one rat (BN64) just moved to a position closer to the reward immediately before pulling the rake, and therefore initially had the rake close to itself; this may have resulted in a correct-direction rate above the chance level (50%) during the test. For instance, perhaps the rat used a strategy of moving closer to the reward (left side) based on the position of the rake handle to try to obtain the reward with its paws and manipulated the rake in the left direction (correct-direction). One of eight rats (BN64) positioned its nose on the side of the handle of the rake opposite to the reward rather than to the same side as the reward (BN57–BN63: *n. s.*; BN64: *p* < 0.01, binomial tests, Supplementary Table 1), and the rat manipulated the rake in the correct-direction significantly more frequently than in the incorrect-direction.

## Discussion

The findings of the present study, in which the modified training procedure was applied, were found to be similar to that of Nagano (2021). The present study showed that one of the eight rats could manipulate a tool according to the position of the food reward without prior experience of obtaining the reward with the tool or perceiving that part of the tool came in contact with the reward. The hypothesis of this study, that the training of motion control improves the tool-manipulation ability in rats, was not supported. The correct-direction rate did not improve within a session after several trials of learning that manipulating the rake in the appropriate direction yielded a reward. In addition, the position of one rat’s (BN64) nose when it first touched the rake was not ipsilateral to the side of the experimental board on which the reward was placed, confirming that the rat did not resort to the simple strategy of moving closer to the reward to try and grab it with its paws before attempting to use the rake to obtain the reward. The rat positioned its nose on the side of the handle of the rake opposite the reward, and a similar tendency was observed in previous rat studies (Nagano and Aoyama, 2017b; Nagano, 2021).

However, it is possible that the rat (BN64) could manipulate the rake in the direction of the reward by chance. Only this rat may have had a behavioral tendency to manipulate the rake in the direction of the reward for some reason (e.g., an innate behavioral tendency to manipulate objects toward foods). It would not be possible that the other seven rats could not manipulate the rake in the correct-direction because, due to their low visual acuity, they could not identify the position of the reward presented on the experimental board (Prusky et al., 2002). In the thread-pulling training, the rats pulled the threads with a reward significantly more often than the thread with no reward. To the best of our knowledge, only the present study and Nagano (2021) have indicated that some animals can manipulate tools according to the position of the target without prior experience of perceiving that one object collided with another object and sent it into motion in controlled experimental settings.

There were some limitations in the present study. It may be possible that the abilities of rodents to manipulate tools appropriately can be detected by conducting the experiments under environmental conditions that match to their biological needs. Rats are nocturnal animals (Norton et al., 1975), but the experiment in the presented study was conducted during the light phase due to the rules of the shared animal room with other researchers in the research institution. Testing the nocturnal rodents during the dark phase would be needed to detect their cognitive abilities more appropriately in future studies (Balcombe, 2010). Moreover, only male rats were used as the subjects in the present studies, as were the previous rat studies (Nagano and Aoyama, 2017a,b; Nagano, 2019a,b, 2021). To testify about the generality of the relationship between the motion control and tool-manipulation, testing with female rats would be needed in future studies. Alternatively, sex differences in tool-use may be observed in rats like capuchin monkeys (*Sapajus libidinosus*) (Falótico et al., 2021). In addition, the experimenter always manipulated the door with her left hand to avoid subconsciously giving the rats cues about the position of the food reward in the positional discrimination test. It may be possible that this procedure created the rats’ side bias, and they manipulated the rake in the same direction in the most of trials.

The changes that the present study made to the experimental procedures used in Nagano (2021) did not enhance the detection of the rodents’ ability to manipulate tools according to food position. It is possible that the tool-manipulation monitoring is not an important factor for tool manipulation in rodents. The procedures in the present study should be conducted in a wider variety of animals to investigate whether tool-manipulation monitoring promotes the subjects’ effective tool-use behavior. Moreover, it can be examined whether the subjects can perceive the reward placed at the same position as the test by conducting the thread-pulling training immediately before the test. In addition, the appropriate distance between the subject and the reward can be determined by applying a procedure similar to the thread-pulling training technique adopted in the present study. Therefore, the procedure in the present study is valuable for investigating tool-use behavior in animals with relatively low visual acuity, such as rats, as well as in animals with relatively high visual acuity.

## Data Availability Statement

The datasets presented in this study can be found in online repositories. The names of the repository/repositories and accession number(s) can be found below: https://osf.io/3x8tz/?view_only=f5c4b90614bd496e8ba6ddd3ed26207a.

## Ethics Statement

The animal study was reviewed and approved by Doshisha University Animal Experiment Committee.

## Author Contributions

AN designed and performed the experiments, made the experimental apparatus, analyzed the data, wrote the manuscript, and prepared the figures.

## Conflict of Interest

The author declares that the research was conducted in the absence of any commercial or financial relationships that could be construed as a potential conflict of interest.

## Publisher’s Note

All claims expressed in this article are solely those of the authors and do not necessarily represent those of their affiliated organizations, or those of the publisher, the editors and the reviewers. Any product that may be evaluated in this article, or claim that may be made by its manufacturer, is not guaranteed or endorsed by the publisher.
