# Supplementary material for: Training of Motion Control May Not Improve Tool-Manipulation Ability in Rats (Rattus norvegicus)
Source: Front Psychol. 2022 Jul 13;13:931957. doi: 10.3389/fpsyg.2022.931957 (PMC9326322; doi:10.3389/fpsyg.2022.931957)
Supplement: Supplementary file 1 [file Data_Sheet_1.docx]

Supplementary Material

# Details of the Apparatus

The experimental box (outer dimensions: 21.0 cm wide × 21.0 cm long × 25.6 cm high) was made from transparent acrylic boards. The box was placed on a desk in the experimental room. Transparent sliding doors (21.0 cm wide × 32.0 cm high × 0.3 cm thick), which the experimenter could open/close by hand, were mounted on the front of the box. One of two kinds of sliding doors (one without a hole and one with a square hole) was always placed in front of the experimental box. The door had a square hole in its upper portion and was used to offer food rewards to the rats by hand. The square hole in the door (1.5 cm wide × 1.5 cm high) was centered horizontally and located at a height of 21.0 cm. An experimental board (23.9 cm wide × 33.5 cm long × maximum 0.8 cm thick), on which the tool and reward were presented, was set in front of the sliding door. The board consisted of a white cutting mat (23.0 cm wide × 32.0 cm long × 0.3 cm thick; Sekisei, Co., Ltd., Osaka, Japan) placed on a transparent acrylic board (23.9 cm wide × 33.5 cm long × 0.5 cm thick). Black lines were drawn in a square lattice on the mat at 1.0-cm intervals. Black drawing paper was laid under the experimental box.

The rake-shaped tool (3.3 cm maximum wide × 6.1 cm long × 3.0 cm high; weight: 4.48 g; Figure 1A) had a rectangular blade (3.5 cm wide × 1.0 cm high × 0.5 cm thick) and a wire handle (0.9 cm maximum wide × 5.5 cm long). The blade was made of a plastic plate covered with resin for dental use (Ostron II Blue, GC Corporation, Tokyo, Japan). A handle made of wire and resin was glued to the center of the blade. A vertical wire (3.0 cm long) pointing upward was glued to each end of the blade to prevent the rats from pulling the rake in the box.

In addition, two kinds of threads were used in the thread-pulling training (Figure 1B). Three-hundred and four threads were used for each kind of thread (608 threads in total). Each thread with a reward (weight: approximately 0.3 g) consisted of three strands of white embroidery thread (COSMO Size 25 Floss, color number: 100; Lecien Corporation, Kyoto, Japan), a piece (one-eighth to one-sixth) of chocolate-flavored loop cereal (Ciscorn Sakusaku Ring, Nissin Cisco Co., Ltd., Osaka, Japan), and a gem clip (6.0 mm maximum wide × 12.0 mm maximum long, color: silver, KOKUYO Co., Ltd., Osaka, Japan). The lengths of the threads were not identical in these two kinds of threads, but these kinds of the threads were made to control the length of the thread part to approximately 5.0 cm.

During the training and the testing sessions, the subject’s behavior was recorded by a video camera (HDC-TM30; Panasonic, Japan) mounted above the experimental box. The experimenter sat in front of the box, observed the subject’s behavior, and performed the following behavioral procedures.

# Details of the Procedure

## Habituation

Before the training phase, the rats were handled for 5 min per day for 5 days. Feeding restriction was introduced to control the subjects’ weight on the third day of handling. From the third day on, each rat was habituated to the food reward by receiving the cereal in its cage for 5 days. The same cereal was used as rewards in the previous tool-use studies with rats (Nagano, 2019a, 2019b, 2021; Nagano and Aoyama, 2017a, 2017b).

## Rake-pulling training

This training consisted of rake-pulling training with and without door manipulation (Figure 2). The rake-pulling training was divided into eight phases (Figure 2).

The sliding door with a hole was used during the rake-pulling training. In Phase 1 of the training without door manipulation, the rake was placed on the experimental board so that the wires glued to either end of the rake blade touched the sliding door (Figures 1A and 2). The sliding door was kept open during this phase so that there was a 1.7-cm gap between the bottom edge of the sliding door and the surface of the experimental board.

At the beginning of the session, the rat was placed in the box. A trial began when the experimenter placed the rake at a defined position on the board (Figure 3A). At the beginning of Phase 1, the trial ended either when the rat touched the rake with its left paw, right paw, nose, or mouth, or when 60 s had passed. If the rat touched the rake within 60 s, then the experimenter retrieved the rake and offered a reward by hand through the small hole in the sliding door (end of the trial; successful trial, Supplementary Video 1). If the rat did not touch the rake within 60 s, then the experimenter retrieved the rake and did not offer a reward (end of the trial; failed trial). The rake was alternately placed on either side of the board; hence, either arrangement was adopted in one-half of the trials during each session in a pseudo-randomized order.

In Phase 1 of the rake-pulling training without door manipulation, the criterion for reward obtainment gradually became stricter. At the beginning of this phase, the experimenter offered a reward when the rat touched the rake with either its paw, nose, or mouth. After the rat consistently pulled the rake in this phase, the experimenter offered a reward only when the rake moved due to the rat touching the rake. Each rat was moved to Phase 2 when it exhibited rake-pulling behavior with its paws or mouth for 10 trials. In all eight phases, successful trials were cumulative and not necessarily consecutive. In Phases 2 to 8, the distance between the blade of the rake and the sliding door of the experimental box was increased by 1.0 cm each phase (Figures 2 and 3A). Over these seven phases, a trial was only recorded as successful when the rat pulled the rake so that one or both of the wires glued to each end of the rake blade touched the sliding door. In Phases 2 to 8, the rat advanced to the next phase after only five successful trials. Training began on each day with Phase 1 if left uncompleted or with the phase reached by the rat in the previous training session. This training continued for every rat until all the rats successfully reached Phase 8 (i.e., seven sessions); six rats surpassed Phase 8 (subject numbers: BN57, BN59, BN60–BN62, and BN64). Additional training sessions were conducted for two rats (BN58: five additional sessions; BN63: three additional sessions).

In the rake-pulling training with door manipulation, the rat was placed in the box at the beginning of the session with the sliding door closed. The rake was placed on the experimental board so that the distance between the rake blade and the door was 7.7 cm—i.e., the same position as in Phase 8 of the rake-pulling training without door manipulation (Figure 3A). The door was opened to create a 1.7-cm gap between the bottom edge of the door and the surface of the experimental board 3 s after the rake was placed on the board (trial start). The experimenter closed the door (end of the trial) either when the rat pulled the rake so that one or both of the wires glued to either end of the rake blade touched the door or when 60 s had passed. The experimenter always manipulated the door with her left hand to avoid subconsciously giving the rats cues about the location of the food reward by which hand the experimenter used for the manipulation of the door. She always used her right hand to press the stopwatch button at the start of each trial. This training continued for every rat until it completed 30 or more successful trials in one session. One rat (BN57) never pulled the rake in any of the trails in Session 1 or in Trials 1 to 15 of Session 2. For this rat (BN57), the experimenter conducted the same procedure as in Phase 5 of the rake-pulling training without door manipulation from Trial 16 in Session 2 of the rake-pulling training with door manipulation. The rat (BN57) reached Phase 8 of the rake-pulling training without door manipulation within this session, and training with door manipulation was conducted on this rat in the next session.

## Rake-manipulation training

The achievement criterion was set for each condition depending on which side of the board the rake was placed on. As in the rake-pulling training, the rake was placed on the right side in half of the trials of a session and on the left side in the other half in a pseudo-randomized order. Session 1 began with Phase 1 under the left side condition or Phase A under the right side condition. Eight successful, consecutive or non-consecutive trials advanced the rat to the next phase in each phase and arrangement condition. Training began in each session with Phase 1 if left uncompleted or with the phase to which the rat had advanced in the previous training session. For the rats that did not advance beyond the first session of a given phase, training in the next session began with the previous phase; e.g., if a rat began Phase 3 when the rake was placed on the left side of the experimental board and performed fewer than eight successful trials, then the rat was considered to have not passed Phase 3, and training regressed to Phase 2 in the next session.

A rat could pass Phase 4 when the rake was placed on the left side but could only reach Phase B when the rake was placed on the right side. The rake-manipulation training continued until the rat achieved the criterion of the last phase under both arrangement conditions (Phases 4 and D) and succeeded in 32 or more trials for two consecutive sessions. The lower limit of this training was 30 sessions, and the upper limit was 60 sessions. In four of the eight rats, fewer sessions were conducted than the upper limit (BN58: 30 sessions, BN61: 48 sessions, BN62: 30 sessions, and BN64: 30 sessions). In the other four rats (BN57, BN59, BN60 and BN63), the training was continued to the upper limit (60 sessions).

## Thread-pulling training

At the beginning of the session, the rat was placed in the box with the sliding door (without a hole) closed. The experimenter opened the door 3 s after placing the thread with or without a reward at a defined position on the board (Figure S2A and S2B). The experimenter closed the door either when the rat pulled the thread to the position in which either the thread with the reward or the knot without a reward entered the box or after 60 s (end of the trial). The experimenter did not retrieve the threads immediately after the rat had finished pulling the thread but recovered them at the end of the session.

The defined position of each thread was set based on the squares of the board (Figure S2A). The threads with a reward or knot were positioned at one area selected randomly from Areas 1–19 (Figure S2A); the gem clip of each thread was placed on the edge of the board (Figure S2B), and the reward, thread, and gem clip were arranged in a straight line (Figure S2B). The rat was presented with a reward thread in half of the trials and a knotted thread in the other half in a pseudo-randomized order. Each type of thread was presented once in each area on the board per session (Figure S2A). The thread-pulling training was performed for two sessions.

## Positional discrimination test

This training used an identical rake to the one used in the rake-pulling and rake-manipulation training (Figure 1A), and a sliding door without a hole was used. At the beginning of the session, the rat was placed in the box with the door closed. The door was opened (trial start) 3 s after the experimenter placed the rake at the center of the experimental board and placed the reward on either the left or right side of the rake (Figure 3C). The reward was positioned so that the blade of the rake would not touch it even if the rat pulled the rake perpendicularly. Thus, the rats could use only the position of the reward in relation to the rake as a cue to determine the correct direction in which to manipulate the rake. The blade of the rake was 7.7 cm from the door and 2.0 cm from the reward. The trial ended either when the rat had obtained the reward within 60 s (successful trial) or after 60 s (failed trial). The daily experimental sessions in this test comprised 40 trials.

# Details of results

## Rake-pulling training

In the rake-pulling training without door manipulation, all rats attained the criterion after 4 (BN62) to 12 (BN58) sessions. In the rake-pulling training with door manipulation, all rats attained the criterion within one session except for one (BN57), which attained it within three sessions.

## Rake-manipulation training

Four of the eight rats (BN58, BN61, BN62, and BN64) satisfied the criteria of the rake-manipulation training after 15 (BN58) to 48 (BN61) sessions, while the other four rats (BN57, BN59, BN60, and BN63) did not meet the criteria within the 60-session limit (Figures S5 and S6). No marked differences in performance were observed between the conditions in which the rake was placed on the left or right side of the board (Figures S5 and S6).

## Thread-pulling training

For the thread-pulling rates, thread type (*F* (1, 7) = 26.50, *p* < 0.001, *η_p_*^2^ = 0.79) and session (*F* (1, 7) = 6.51, *p* < 0.05, *η_p_*^2^ = 0.48) had significant effects on success, and there was a significant interaction between thread type and session (*F* (1, 7) = 7.20, *p* < 0.05, *η_p_*^2^ = 0.51) (Figure S7A). Subsequent simple main effect analyses revealed that the rats pulled the threads with a reward significantly more than the threads without one in Sessions 1 and 2 (Session 1: *F* (1, 7) = 18.78, *p* < 0.01, *η_p_*^2^ = 0.73; Session 2: *F* (1, 7) = 18.86, *p* < 0.01, *η_p_*^2^ = 0.73). In addition, the rats pulled the threads without a reward significantly more frequently in Session 1 than 2 (*F* (1, 7) = 7.00, *p* < 0.05, *η_p_*^2^ = 0.50), but they pulled the threads with a reward at the same frequency in both (*F* (1, 7) = 1.00, *n. s.*, *η_p_*^2^ = 0.13).

Similarly, in the thread-contacting rates, thread type (*F* (1, 7) = 16.96, *p* < 0.01, *η_p_*^2^ = 0.71) and session (*F* (1, 7) = 6.23, *p* < 0.05, *η_p_*^2^ = 0.47) had significant effects on success, and there was a significant interaction between thread type and session (*F* (1, 7) = 6.86, *p* < 0.05, *η_p_*^2^ = 0.50) (Figure S7B). Subsequent simple main effect analyses revealed that the rats contacted the threads with a reward significantly more than the threads without a reward in Sessions 1 and 2 (Session 1: *F* (1, 7) = 11.29, *p* < 0.05, *η_p_*^2^ = 0.62; Session 2: *F* (1, 7) = 14.32, *p* < 0.01, *η_p_*^2^ = 0.67). In addition, the rats came in contact with the threads without a reward significantly more frequently in Session 1 than in 2 (*F* (1, 7) = 6.59, *p* < 0.05, *η_p_*^2^ = 0.13), but they came in contact with the threads with a reward at the same level of frequency in both (*F* (1, 7) = 1.00, *n. s.*, *η_p_*^2^ = 0.49). The results in the thread-pulling training indicate that the rats could discriminate between the threads with and without a reward.


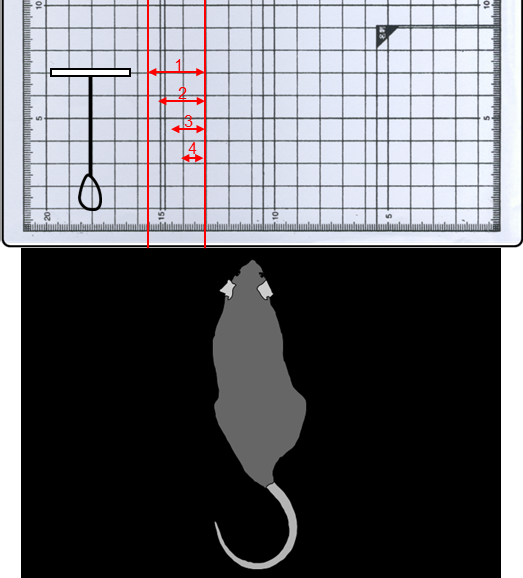


**A**


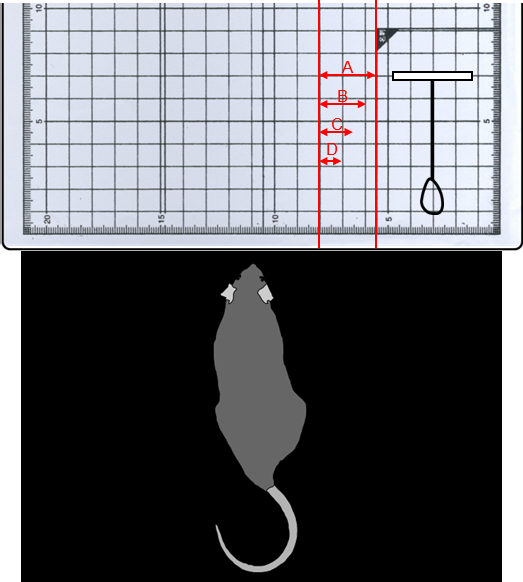


**B**

**Supplementary Figure 1.** Criterion ranges for each phase of the rake-manipulation training. **(A)** Red lines show the successive criterion ranges from Phases 1 and 4 when the rake was placed on the left side of the experimental board from the rats’ perspective. **(B)** Red line shows the successive criterion ranges from Phases A to D when the rake was placed on the right side of the board from the rats’ perspective.


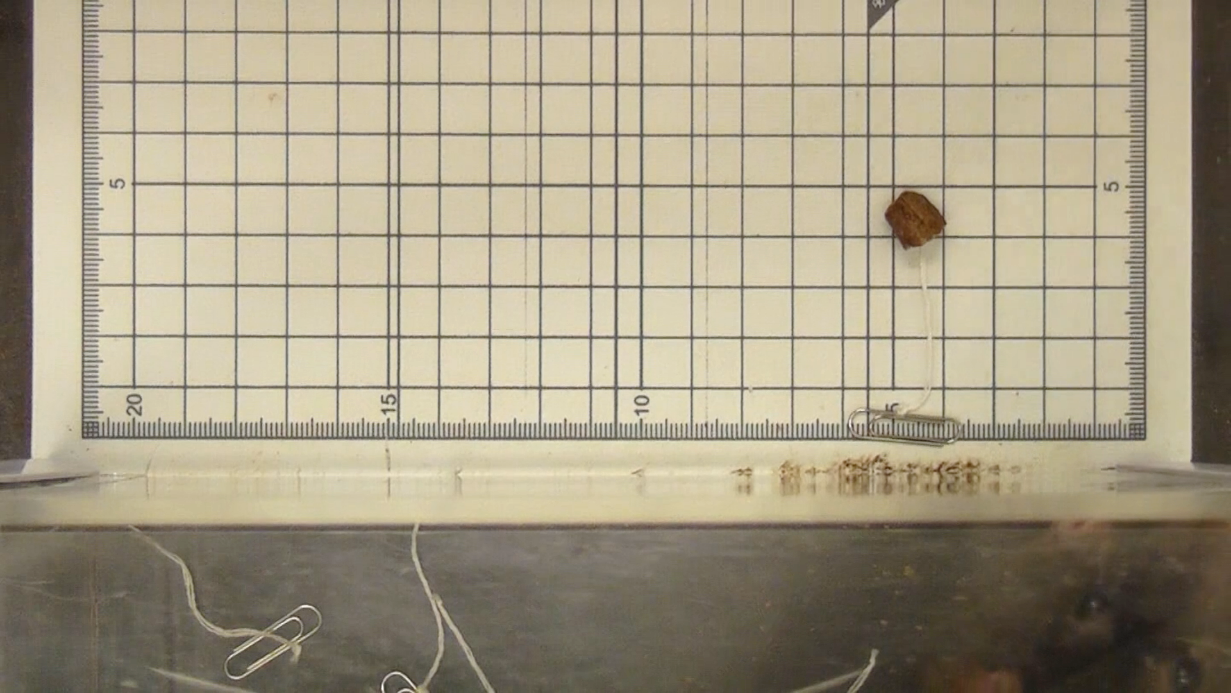


**A**

Food reward

Embroidery thread

Gem clip


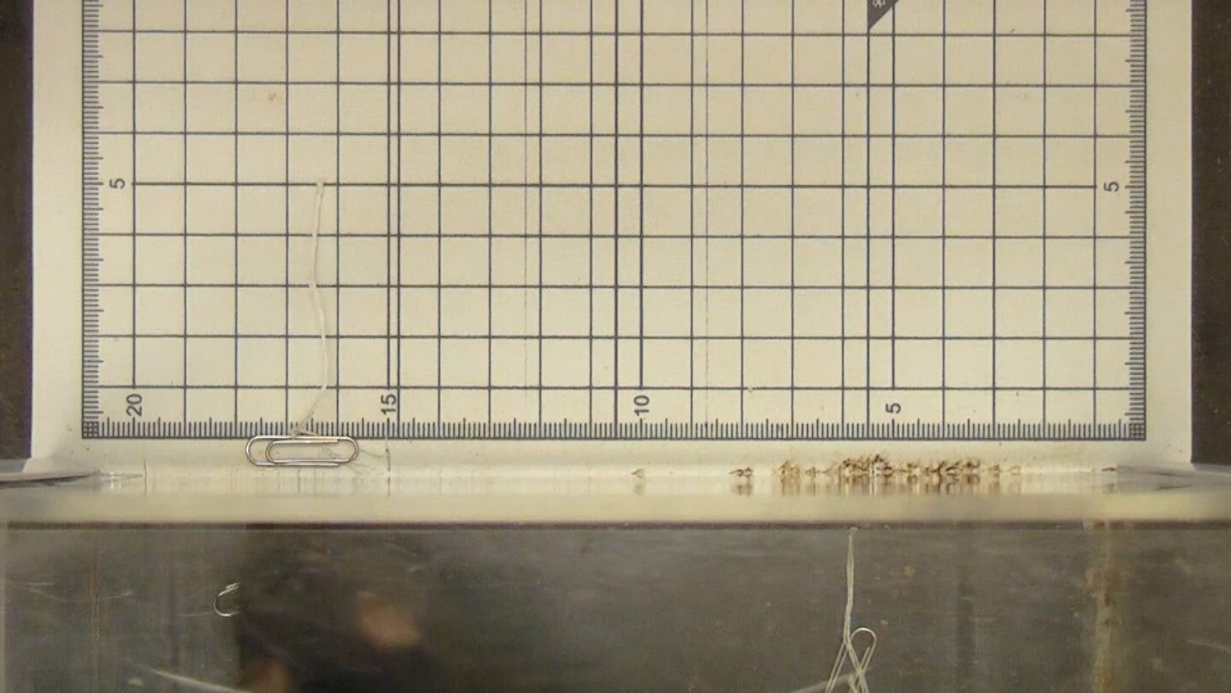


**B**

**C**


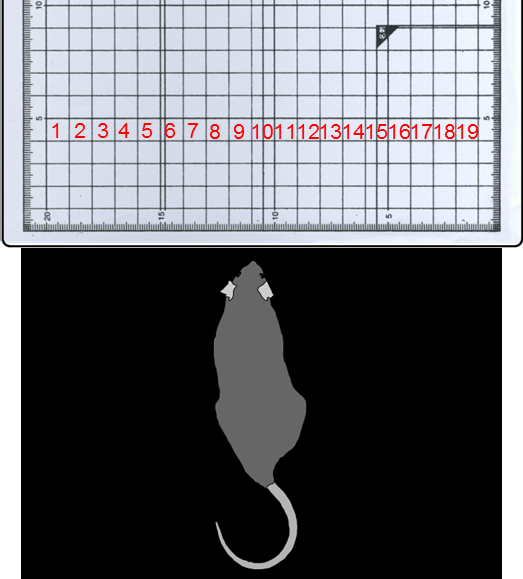


**Supplementary Figure 2.** Arrangement of the thread with or without a reward in the thread-pulling training. **(A)** Example of the arrangements of the thread with a reward. **(B)** Example of the arrangements of the thread without a reward. **(C)** The positions of the reward on the thread with a reward or the knot on the thread without a reward. The reward or knot was positioned in one area selected randomly from Areas 1–19.


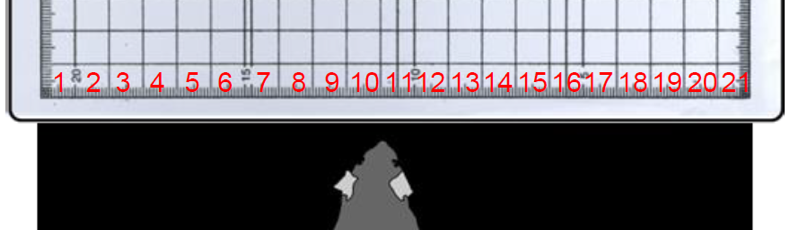

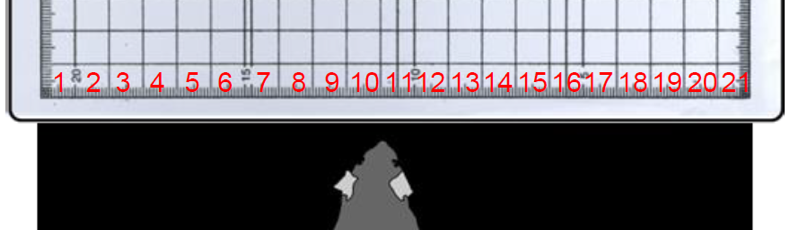


**BN57**

**No-reward**

**No-reward**

**Reward**

**Reward**

**BN58**

**BN59**

**BN60**

**Supplementary Figure 3.** Individual (BN57–BN60) results for the number of trials in which the rat’s nose was located at each area in each trial in the positional discrimination test. The left panel indicates the results in which the reward was placed on the right side of the rake; the right panel indicates the results in which the reward was placed on the left side of the rake. Each broken line indicates the position of the handle of the rake.


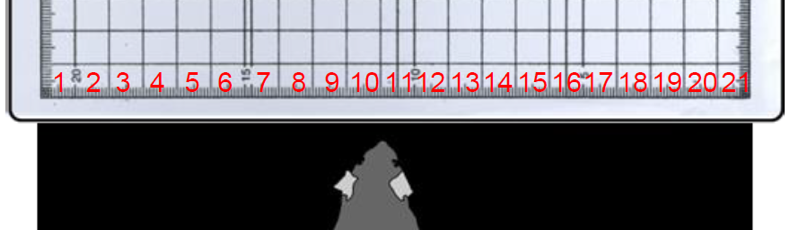

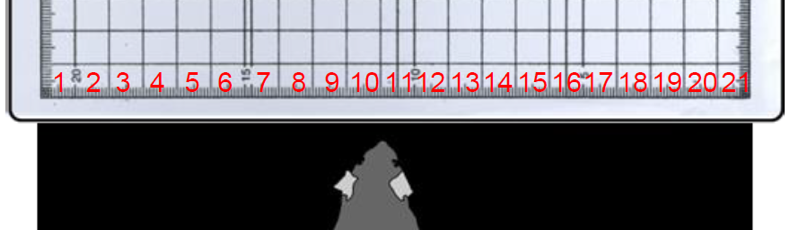


**BN61**

**No-reward**

**No-reward**

**Reward**

**Reward**

**BN62**

**BN63**

**BN64**

**Supplementary Figure 4.** Individual (BN61–BN64) results for the number of trials in which the rat’s nose was located at each area in each trial in the positional discrimination test. The left panel indicates the results in which the reward was placed on the right side of the rake; the right panel indicates the results in which the reward was placed on the left side of the rake. Each broken line indicates the position of the handle of the rake.

**
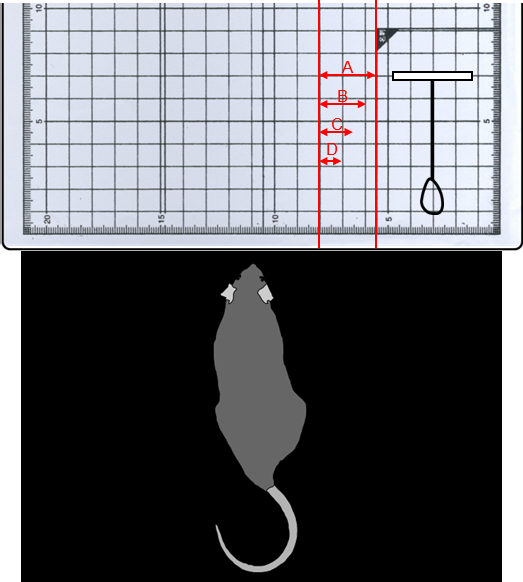

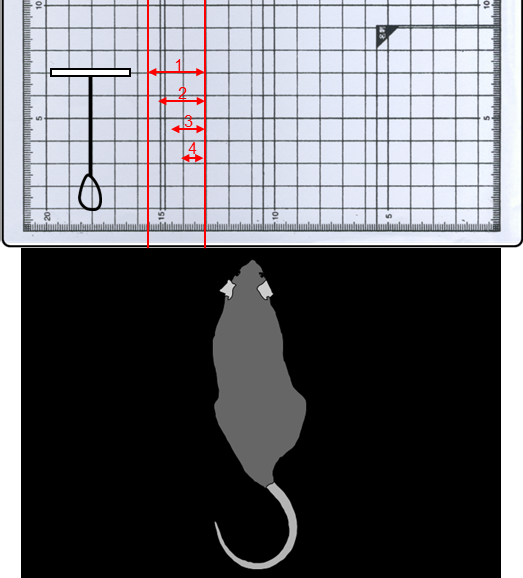
**

**BN57**

**BN58**

**BN59**

**BN60**

**Supplementary Figure 5.** Individual (BN57–BN60) success rates across trials in each arrangement of the rake-manipulation training. The left panel corresponds to the condition under which the rake was placed on the left side of the experimental board; the right panel corresponds to the condition under which the reward was placed on the right side of the board. The lower numbers on each horizontal axis indicate the phase at the start of the session. Some rats (BN59, BN60) returned to the previous phase over the course of training. Extra training was provided to one rat (BN58).

**
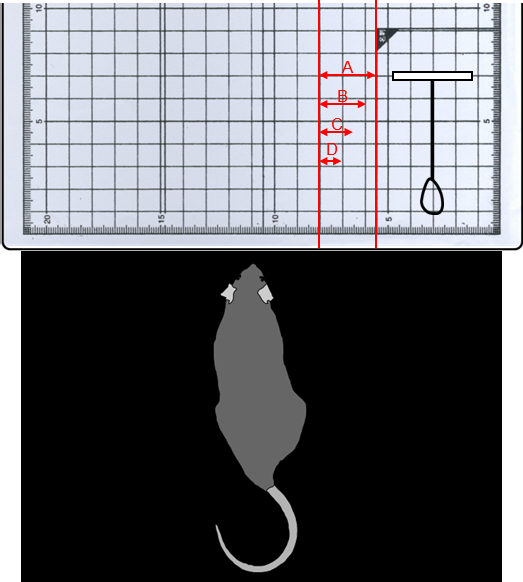

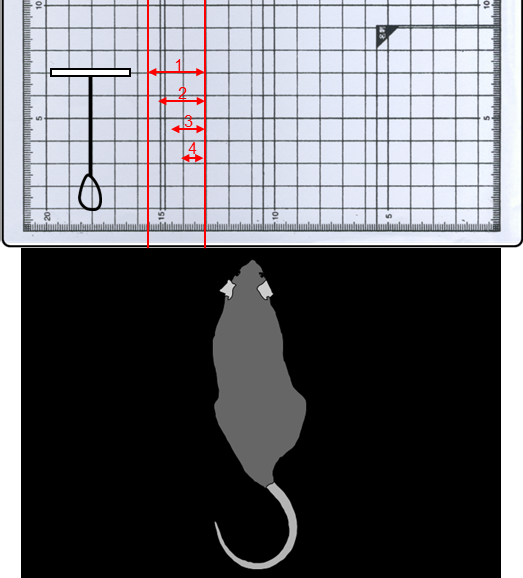
**

**BN61**

**BN62**

**BN63**

**BN64**

**Supplementary Figure 6.** Individual (BN61–BN64) success rates across trials in each arrangement of the rake-manipulation training. The left panel corresponds to the condition under which the rake was placed on the left side of the experimental board; the right panel corresponds to the condition under which the reward was placed on the right side of the board. The lower numbers on each horizontal axis indicate the phase at the start of the session. Some rats (BN63, BN64) returned to the previous phase over the course of training. Extra training was provided to two rats (BN62, BN64).

**

**

**A**

*

*

*

**

**B**

**Supplementary Figure 7.** Performance in the thread-pulling training. **(A)** Average thread-pulling rates per session. **(B)** Average thread-contacting rates per session. The error bars indicate standard errors. ^*^ *p* < 0.05, ^**^ *p* < 0.01, ^***^ *p* < 0.001

**BN58**

**BN57**

**BN60**

**BN59**

**BN61**

**BN62**

**BN63**

**BN64**

**Supplementary Figure 8.** Individual (BN57–BN64) changes in the average correct-direction rate in the positional discrimination test. The broken line indicates the level of chance. Trials 31 to 35 of one rat (BN58) and 26 to 40 of another (BN59) were excluded from the analysis, as the rats never pulled the rake in these trials.

**Supplementary Table 1.** Individual rates of the ipsilateral trials of the positional discrimination Test. The cell filled with grey shows the ipsilateral trial rates for the rat that manipulated the rake in the correct-direction significantly more frequently than in the incorrect-direction. ^**^ *p* < 0.01

| Subject number | Rate of ipsilateral trials (%) |
| --- | --- |
| BN57 | 54.55 |
| BN58 | 55.56 |
| BN59 | 52.38 |
| BN60 | 47.50 |
| BN61 | 50.00 |
| BN62 | 52.94 |
| BN63 | 48.57 |
| BN64 | 30.00^**^ |
